# Exploiting the anti-fibrotic effects of statins on thoracic aortic aneurysm progression: results from a meta-analysis and experimental data

**DOI:** 10.3389/fphar.2024.1426982

**Published:** 2024-08-01

**Authors:** Veronika A. Myasoedova, Sara Rega, Vincenza Valerio, Donato Moschetta, Ilaria Massaiu, Giorgia Bonalumi, Giampiero Esposito, Valentina Rusconi, Francesca Bertolini, Gianluca Lorenzo Perrucci, Paolo Poggio

**Affiliations:** ^1^ Centro Cardiologico Monzino Istituto di Ricovero e Cura a Carattere Scientifico (IRCCS), Milan, Italy; ^2^ Department of Biomedical, Surgical and Dental Sciences, University of Milan, Milan, Italy

**Keywords:** statins, thoracic aortic aneurysm, fibrosis, vascular smooth muscle cells, outcomes

## Abstract

**Aims:**

Thoracic aortic aneurysm (TAA) that progress to acute aortic dissection is often fatal and there is no pharmacological treatment that can reduce TAA progression. We aim to evaluate statins’ effects on TAA growth rate and outcomes using a meta-analysis approach.

**Methods and results:**

A detailed search related to the effects of statins on TAA was conducted according to PRISMA guidelines. The analyses of statins’ effects on TAA growth rate were performed on 4 studies (n = 1850), while the impact on outcomes was evaluated on 3 studies (n = 2,867). Patients under statin treatment showed a reduced TAA growth rate (difference in means = −0.36 cm/year; 95%CI: −0.64, −0.08; *p* = 0.013) when compared to controls, patients not taking statins. Regarding the outcomes (death, dissection, or rupture of the aorta, and the need for operative repair), statins exhibited a protective effect reducing the number of events (log odds ratio = −0.56; 95%CI: −1.06, −0.05; *p* = 0.030). *In vitro*, the anti-fibrotic effect of atorvastatin was tested on vascular smooth muscle cells (VMSC) isolated from patients with TAA. Our results highlighted that, in transforming growth factor beta 1 (TGF-β1) pro-fibrotic condition, VSMC expressed a significant lower amount of collagen type I alpha 1 chain (COL1A1) when treated with atorvastatin (untreated = +2.66 ± 0.23 fold-change vs. treated = +1.63 ± 0.09 fold-change; *p* = 0.014).

**Conclusion:**

Statins show a protective effect on TAA growth rate and adverse outcomes in patients with TAA, possibly via their anti-fibrotic properties on VSMC. Given the current lack of effective drug treatments for TAA, we believe our findings highlight the need for more in-depth research to explore the potential benefits of statins in this context.

## Introduction

Thoracic aortic aneurysm (TAA) is a chronic disease characterized by localized irreversible dilatation that can progress to life-threatening consequences, such as acute aortic dissections with subsequent rupture of the aorta, which are often fatal (Liu et al., 2020; Cho et al., 2023). Numerous risk factors, such as genetic predisposition, smoking, uncontrolled hypertension, and dyslipidemia, lead to destructive remodeling of the aortic wall resulting in adverse outcomes (Zhou et al., 2022). So far, no effective drug treatment has been found and open repair remains the gold standard for aortic aneurysm treatment (Hong and Coselli, 2021).

Statins are known for their pleiotropic effects. They are not only effective in correcting lipid imbalances but also enhance the overall function of vascular endothelium (Zhao et al., 2023). Additionally, statins reduce inflammation and subsequent oxidative stress, and stabilize atherosclerotic plaques (German and Liao, 2023). Indeed, recent evidence suggests that statins reduce inflammation through various mechanisms, including reducing C-reactive protein and modulating multiple cellular pathways, such as Ras/Rho, nuclear factor-κB and activator protein-1-mediated pro-inflammatory pathways, and nuclear factors such as peroxisome proliferator-activated receptor and Kruppel-like factor-2 (Jain and Ridker, 2005).

Statins also play a role in preventing the development of tissue fibrosis, which has been supported by retrospective analyses of large patient groups and studies in animals (Dolivo et al., 2023). Experimental evidence supports the anti-fibrotic potential of statins. Specifically, screening for YAP inhibitors has identified statins as modulators of fibrosis (Santos et al., 2020). Additionally, statins may attenuate fibrosis by suppressing iNOS expression and the CTGF (CCN2)/ERK signalling pathway (Zhu et al., 2013). Furthermore, it is known that statins inhibit growth factor expression and modulate pro-fibrogenic markers in fibroblasts (Watts et al., 2005). While the impact of statins on TAA is not well-established, some research indicates that statins may slow the progression of TAA by affecting the rate at which the thoracic aorta expands (Jovin et al., 2012; Stein et al., 2013; Angeloni et al., 2015; Masaki et al., 2018). Moreover, these studies suggest that patients with TAA on statin therapy experience better outcomes (Jovin et al., 2012; Stein et al., 2013; Angeloni et al., 2015).

Thus, we hypothesized that statin therapy may have a protective effect on TAA progression and adverse outcomes probably due to antifibrotic properties. To this purpose we conducted a thorough meta-analysis to: i) explore the relationship between statin therapy and the progression and complications of TAA and ii) investigate whether statins influence adverse outcomes such as death, dissection, or rupture of the aorta, and the need for operative repair in TAA patients. Additionally, we examined the anti-fibrotic effects of statins on vascular smooth muscle cells (VSMC) derived from TAA patients in an *in vitro* setting.

## Materials and methods

### Data sources and searches

To specify the objectives, study selection criteria, outcomes, and statistical methods a detailed protocol for the search strategy of this review was developed prospectively. All available studies on the association between the effect of statins and thoracic aortic aneurysm (TAA) were evaluated through a systematic search of electronic databases (PubMed, Web of Science, and Scopus) according to the Preferred Reporting Items for Systematic Reviews and Meta-Analyses (PRISMA) guidelines (Page et al., 2021). Following search string was applied: (thoracic aortic aneurysm OR TAA OR ascending aortic aneurysm OR Asc aortic aneurysm OR thoracic aortic growth rate) AND (statin). The last search was performed in June 2023. Moreover, the reference lists of all included articles were manually consulted. Two authors (VAM and GLP) analyzed each article and separately performed the data extraction. In case of disagreement, a third investigator was consulted (PP). Discrepancies were resolved by consensus.

### Study selection, data extraction, and quality assessment

According to the stipulated protocol, all studies reporting data about the association between the effect of statins on TAA growth rate and outcomes in TAA patients were included. Case reports, reviews, and articles on animal models were excluded. In each study, data regarding major clinical and demographic characteristics and outcomes in patients with TAA were extracted. The evaluation of the methodological quality of each study was performed accordingly Newcastle–Ottawa Scale (NOS). The scoring system encompasses three major domains (selection, exposure, outcome) and a resulting score range between 0 and 9, a higher score representing a better methodological quality.

### Patients’ enrollment for aortic smooth muscle cell isolation

Aortic vascular smooth muscle cell (VSMC) were isolated from thoracic aortic tissues collected in the surgery room from patients with sporadic (*i.e.,* non-genetically determined) thoracic aortic aneurysm (TAA). The study protocol followed the principles of the Declaration of Helsinki. The “Ethics Committee of the IRCCS Centro Cardiologico Monzino” approved the study protocol (CCM1462/RE3001). All enrolled patients signed written informed consent. Participants also consented to share their de-identified information. We enrolled patients with tricuspid aortic valve candidates to surgery affected by sporadic TAA (n = 6). The preoperative inclusion criteria were the dilation of ascending thoracic aorta, needing for an elective and isolated surgical procedure, in subjects over 18 years of age, of both sexes, without (or undiagnosed) genetic syndrome. Patients previously undergone surgical procedures on aorta, with premature menopause and/or osteoporosis, previous aortic or mitral valve surgery, rheumatic heart disease, active malignancy, liver or kidney chronic failure, thyroid system diseases, positivity for HIV, HCV, HBsAg, SARS-CoV-2, and chronic or acute inflammatory states (sepsis, autoimmune disease, and inflammatory bowel disease) were excluded.

### Aortic vascular smooth muscle cells isolation and culture

Isolation of aortic VSMC from patients’ aortic walls was performed following a previously described method (Perrucci et al., 2020). Briefly, surgical samples of aortic tunica media were washed with phosphate buffer saline (PBS, Euroclone, Milano, Italy), minced and digested overnight (O/N) at 37°C in a solution of 2 mg/mL collagenase type II (Worthington Biochemical Corporation, Lakewood, NJ, United States) in complete Smooth Muscle Cell Growth Medium-2 (SmGM™-2, Lonza, Basel, Swiss), supplemented with Smooth Muscle Growth Supplement (SmGM™-2 BulletKit™, Lonza, Basel, Swiss). Then, the result of tissue digestion was filtered with 100 μm cell strainer, pelleted and plated in complete SmGM™-2 medium. All the experiments were performed on cultured cells between their second and sixth passage, grown in complete SmGM™-2 medium then treated with 5 ng/mL TGF-β1 (Thermo Fisher Scientific, Waltham, Massachusetts, United States) and 6.6 µM Atorvastatin (United States Pharmacopeia, Rockville, Maryland, United States).

### Immunostaining assay

Patients’ VSMC were plated on 96-well plates, treated with TGF-β1 and Atorvastatin for 48 h with 95% humidity and 5% CO_2_. Then, medium was removed and cells were washed with PBS and then fixed for about 15 min in a solution of 4% paraformaldehyde (PFA, D.b.a. Italia, Segrate, Italy). After that, the primary unconjugated antibody for human COL1A1 (Cell Signaling Technology, Danvers, Massachusetts, United States) was incubated O/N at 4°C. Then, the goat anti-rabbit secondary antibody conjugated with AlexaFluor488 (Thermo Fisher Scientific, Waltham, Massachusetts, United States) was incubated for 1 h at room temperature (RT). As a negative control, species- and isotype matched IgGs were incubated in place of the primary antibodies. 96-well plates were observed and automatically analyzed with Operetta (Revvity, Milano, Italy).

### Western blot analysis

Patients’ aortic VSMC were lysed in cell lysis buffer (Cell Signaling Technology, Danvers, Massachusetts, United States) supplemented with protease and phosphatase inhibitor cocktails (Sigma Aldrich, Burlington, Massachusetts, United States). Total protein extracts were subjected to SDS-PAGE and transferred onto a nitrocellulose membrane. The membranes were blocked for 1 h at RT in 5% non-fat dry milk in Wash Buffer (Tris Buffer Sulfate 1X, 0.1% Tween 20; Sigma Aldrich, Burlington, Massachusetts, United States) and then incubated O/N at 4°C with primary antibodies against human COL1A1 and GAPDH (Cell Signaling Technology, Danvers, Massachusetts, United States). The membranes were incubated with peroxidase-conjugated secondary antibodies (GE Healthcare, Chicago, Illinois, United States) for 1 h at RT. Signals were visualized using the chemiluminescence Western blotting detection system (LiteUP, Euroclone, Milano, Italy). Images were acquired with the ChemiDoc system (Bio-Rad, Hercules, California, United States), and densitometric analysis of membranes was performed using the Image Lab software (Bio-Rad, Hercules, California, United States). In order to normalize, GAPDH was used as a total protein loading control.

### Statistical analysis and risk of bias assessment

Statistical analysis was performed using Comprehensive Meta-analysis Version 3.3.070 (Biostat, Englewood, NJ 2014). The differences in continuous variables were expressed as a standardized mean difference (SDM) and 95% confidence intervals (CI). The differences among cases and controls in outcomes were expressed as odds ratio (OR) with pertinent 95% CI. The overall effect was tested using Z-scores and significance was set at *p* < 0.05. Statistical heterogeneity among studies was assessed with chi-square Cochran’s Q test and with I2 statistic, which measures the inconsistency across study results and describes the proportion of total variation in study estimates, that is due to heterogeneity rather than sampling error. In detail, I^2^ values of 0% indicate no heterogeneity, 25% low, 25%–50% moderate, and 50% or more high heterogeneity (Higgins et al., 2003).

Egger’s test and funnel plots of the logit event rate vs. the standard error was used as a graphical representation to evaluate the risk of bias. To assess the small-study effect, funnel plots were visually inspected for asymmetry and Egger’s test was used to assess publication bias, over and above any subjective evaluation, with *p* < 0.05 being considered statistically significant (Sterne et al., 2001).

In order to be as conservative as possible, the random-effect method was used for all analyses to consider the variability among the included studies. In the case of significant publication bias, Duval and Tweedie’s trim and fill method was used to allow for the assessment of adjusted effect size (Di Minno et al., 2017).

The analyses for the *in vitro* assay results have been performed with GraphPad Prism 9. For the analysis among the groups, the one-way ANOVA was performed, whereas the comparisons between groups were performed with the Tukey *post hoc* test. For both tests *p* < 0.05 was considered statistically significant.

## Results

### Meta-analysis of statin effect on TAA growth rate and outcomes

**FIGURE 1:**
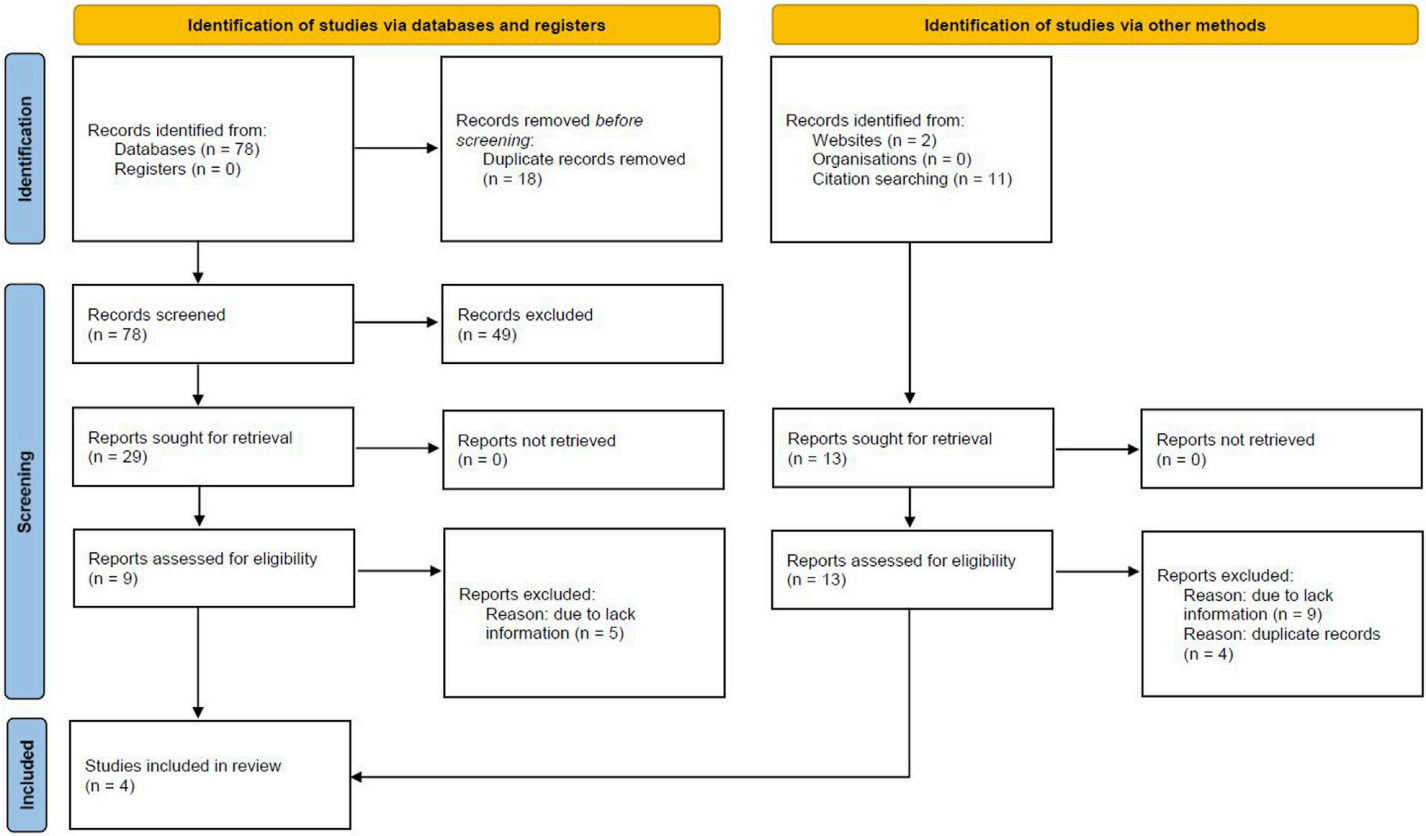
Prisma Flow Chart. The flow chart represents the number of studies evaluated according to PRISMA guidelines.

The search strategy identifies 78 articles, duplicate results were excluded, and after a screening of the titles and the abstracts, nine articles were selected for full-text evaluation. The revision of full-length articles allowed the exclusion of five studies due to irrelevant information in their content. Overall, four studies (Jovin et al., 2012; Stein et al., 2013; Angeloni et al., 2015; Masaki et al., 2018), enrolling 1,850 patients were included in the qualitative and quantitative analyses of the effect of statins on TAA growth rate, while three studies (Jovin et al., 2012; Stein et al., 2013; Angeloni et al., 2015) were analyzed for the impact of statins on outcomes, in 2,867 TAA patients (Figure 1). A total of 67% of included patients were males with a mean age of 66 years (range: 63–70 years). Mean hypertension and mean diabetes were present in 65% and 8% of patients, respectively. The mean follow-up was 6.5 years. Results of the NOS quality assessment are reported in Supplementary Table S1.

**FIGURE 2:**
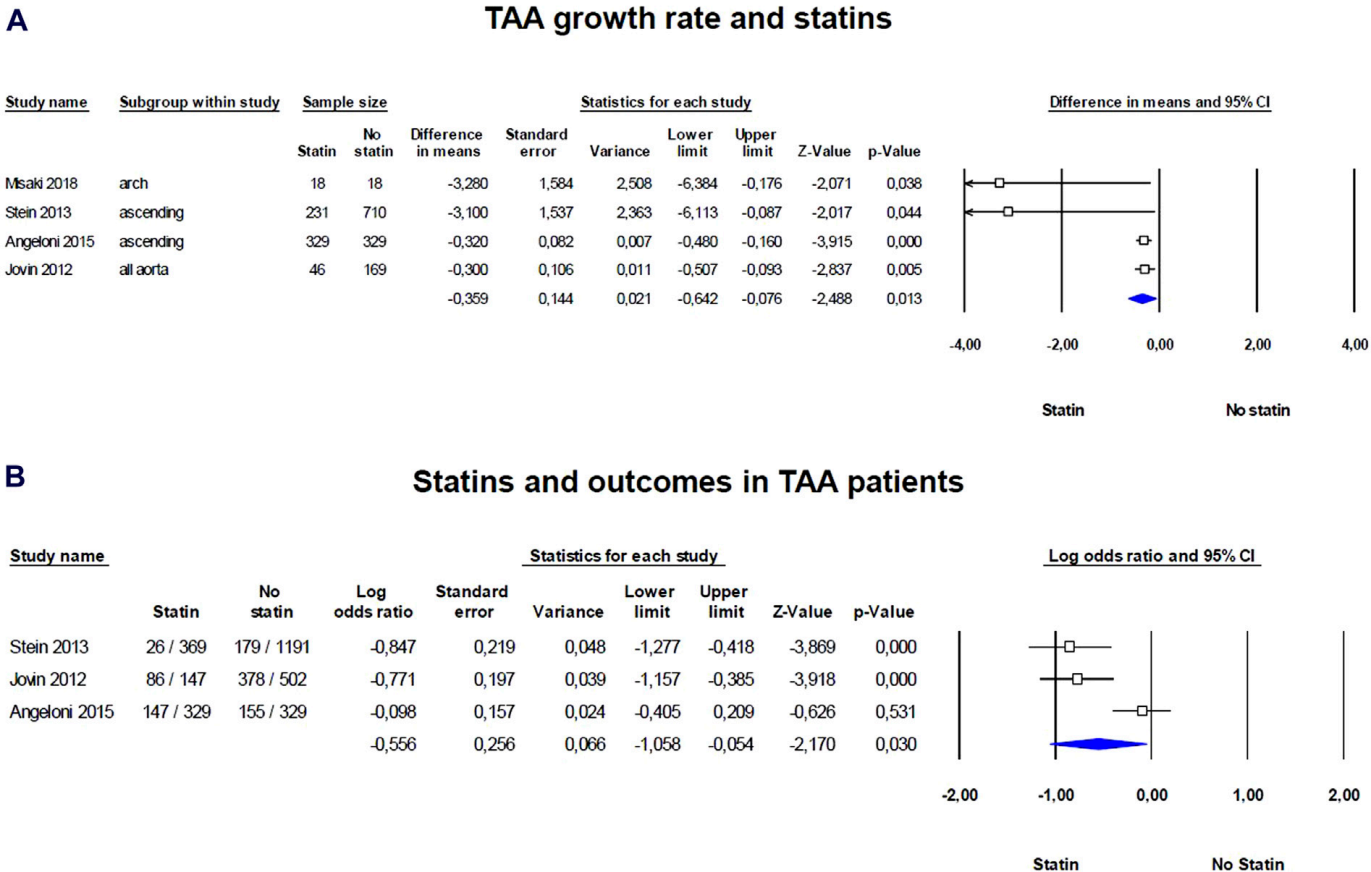
Forest plots of the statin effect on thoracic aorta aneurism (TAA) growth rate and outcomes in TAA patients. **(A)** TAA grown rate was evaluated with the standardized difference in means (SMD) between baseline and follow-up, **(B)** while the effect of statins on outcomes was evaluated with log odds ratio (logOR). The diamond represents the estimated overall effect, while the squares represent each study with its 95% CI.

We observed significant positive effect of statins on TAA growth rate, in particular patients taking statins compared with controls shows significant reduced growth rate SDM of −0.359 (95% CI = −0.642, −0.076; *p* = 0.013; Figure 2A; Supplementary Table S2). The heterogeneity among the studies was relatively high but not significant (I^2^ = 56%; *p* = 0.079). Visual inspection of funnel plots suggested the presence of a significant publication bias confirmed by the Egger’s test (*p* = 0.018; Supplementary Figure S1A). Results were adjusted by means of the Duval and Tweedie trim‐and‐fill method and the difference between statins and control treatment was confirmed (SDM = −0.346; 95% CI = −0.690, −0.002). Thus, statins significantly reduced TAA growth rate with a persistent effect even after adjusting for publication bias.

Regarding outcomes, such as death, dissection, or rupture of the aorta, and the need for operative repair, statins exhibit a protective effect, reducing the number of events in patients with TAA (logOR = −0.556; 95% CI: −1.058, −0.054; *p* = 0.030; Figure 2B; Supplementary Table S3). The heterogeneity among the studies was high and significant (I^2^ = 82%, *p* = 0.004) and the examination of funnel plots of effect size versus standard error was rather symmetrical, thus excluding the presence of any publication bias (Egger’s test *p* = 0.148; Supplementary Figure S1B). Thus, statins were associated with a protective effect against serious aortic events in TAA patients, without evidence of publication bias. Thus, statins appear to have a persistent positive effect on reducing the growth rate of TAAs as well as on outcomes, and they could be considered as part of the management strategy for patients with TAAs.

### Atorvastatin mitigates pro-fibrotic effects of TGF-β1 in patients derived aortic VSMC.

**FIGURE 3:**
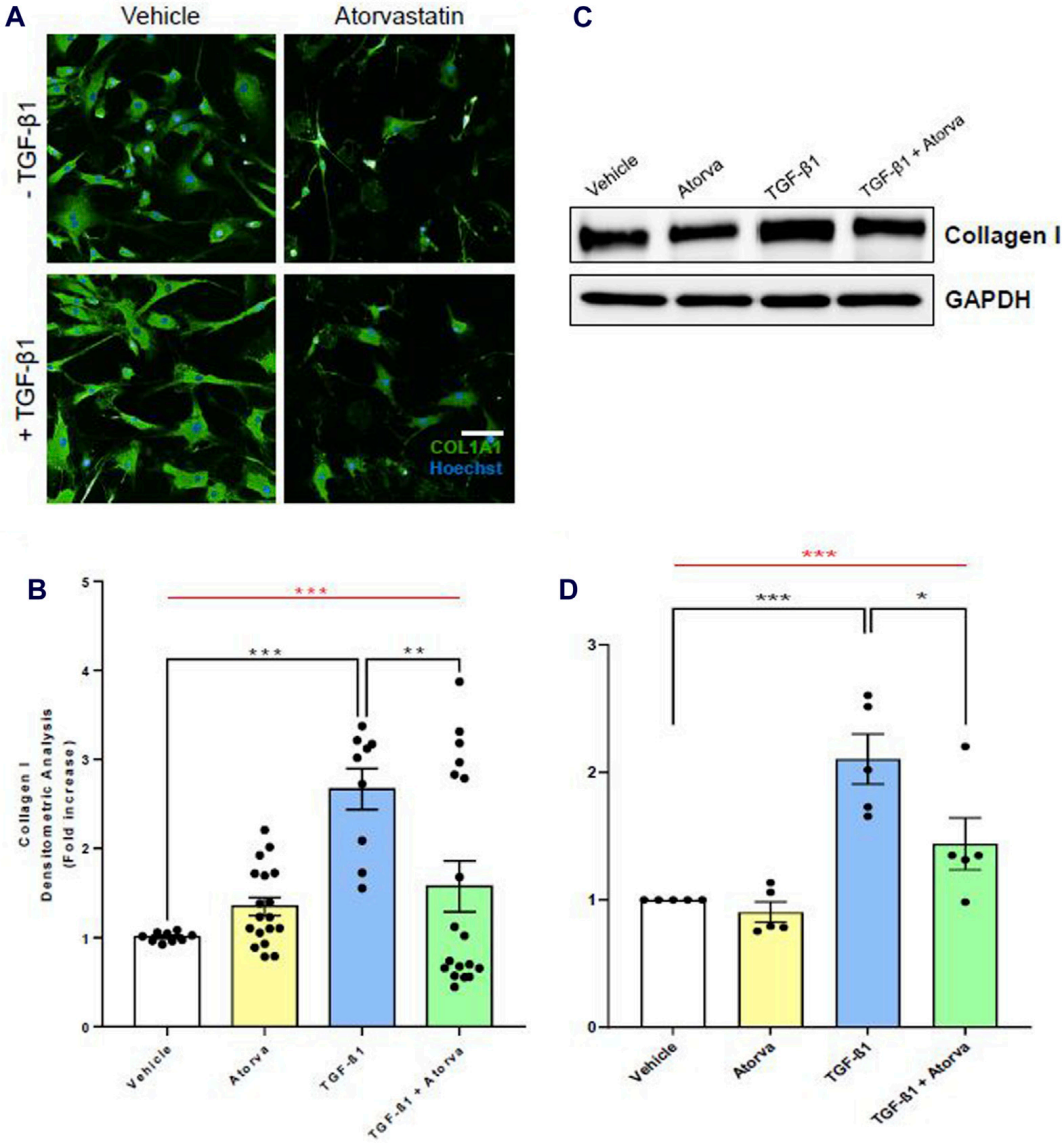
*In vitro* atorvastatin treatment of human aortic VSMC limits collagen I expression in pro-fibrotic conditions. **(A)** Representative images of immunofluorescence assay for COL1A1 (green) on human aortic VSMC in presence or absence of TGF-β1. The nuclei have been stained with Hoechst (in blue). Scale bar = 200 µm. Magnification = ×20. **(B)** Immunofluorescence densitometric quantification of VSMC treated with vehicle (white bar), atorvastatin (Atorva, yellow bar), TGF-β1 (blue bar), and TGF-β1 + atorvastatin (green bar). Collagen I quantification data are shown as mean ± SEM, n = 3 (without atorvastatin) and n = 6 (with atorvastatin). One-way ANOVA: ***p = 0.0001 (red line). Tukey’s multiple comparison post-test: **p < 0.01; ***p = 0.0001. **(C)** Western blot images of COL1A1 expression levels in total protein extracts of human aortic VSMC. GAPDH has been used as loading control. **(D)** Western blot relative densitometric quantification of VSMC treated with vehicle (white bar), atorvastatin (Atorva, yellow bar), TGF-β1 (blue bar), and TGF-β1 + atorvastatin (green bar). Data are shown as mean ± SEM, n = 5. One-way ANOVA: **p < 0.01 (red line). Tukey’s multiple comparison post-test: *p < 0.05.

To evaluate the efficacy of statins in reducing pro-fibrotic events leading to TAA *in vitro*, we treated aortic VSMC of TAA patients with atorvastatin, both in presence and absence of TGF-β1. The pro-fibrotic effect of TGF-β1 at the concentration of 5 ng/mL on VSMC have been frequently reported by literature (Perrucci et al., 2018a; Perrucci et al., 2020). By a high-throughput analysis of an immunofluorescence assay, we show that atorvastatin treatment in TGF-β1-mediated pro-fibrotic condition led to a significant decrease of COL1A1 expression levels (+1.57 ± 0.28 FC in VSMC treated with TGF-β1 + Atorva vs. +2.67 ± 0.23 FC in VSMC treated with only TGF-β1, *p* = 0.0081; Figures 3A, B). We further confirmed this result with a semi-quantitative Western blot assay. Indeed, VSMC treated with TGF-β1 + Atorvastatin had a +1.17 ± 0.20 FC collagen type I increment, whereas VSMC treated with only TGF-β1had a +3.73 ± 1.17 FC collagen I increment (*p* = 0.0358; Figures 3C, D), showing that atorvastatin significantly limits TGF-β1-mediated fibrosis in VSMC derived from TAA patients. Of note, VSMC treated with only Atorvastatin (yellow bars, Figure 3) did not show any statistical difference when compared with cells treated with vehicle (white bars). These results suggest that atorvastatin effectively mitigates the pro-fibrotic processes induced by TGF-β1 in aortic VSMCs derived from TAA patients.

## Discussion

Our meta-analysis highlights the significant role of statins in slowing the growth rate of TAA and improving patient outcomes. Whereas, *in vitro* results supported these data, showing that atorvastatin effectively reduces the pro-fibrotic effects of TGF-β1 in VSMC isolated from TAA patients. This suggests a promising therapeutic avenue for managing TAA progression and complications.

In the last 40 years, studies focused on the research of an effective therapeutic tool for TAA unveiled how much demanding this challenge is. Although several mechanisms of TAA are nowadays well-known (Rega et al., 2023), a complete understanding of this pathological condition is still missing, and an effective treatment is yet to be found.

The current body of research has identified numerous biomarkers and microscopic changes in aortic tissue that could play a role in the development or prediction of TAA (Schlatmann and Becker, 1977), a leading cause of lethal aortic dissections (Peterss et al., 2016). Alterations in the aortic wall can impact all layers, including the intima, media, and adventitia. However, the most harmful changes are the fragmentation of elastin fibers and fibrosis in the media layer, which disrupt the balance of the aortic wall’s structure and function (Schlatmann and Becker, 1977). The excessive buildup of collagen by the VSMC in the media layer often leads to the breakdown of the lamellar structure, the functional unit of the aorta that provides strength and elasticity. These pro-fibrotic events weaken the aortic wall, contributing to the formation of TAA (Peterss et al., 2016; Perrucci et al., 2020).

The secretion of collagen by VSMCs is due to a harmful transdifferentiation of these cells from a contractile to a synthetic/secretory phenotype, triggered by TGF-β, a key pro-fibrotic mediator. TGF-β is a critical regulator of various cellular functions, including growth, proliferation, and differentiation (Perrucci et al., 2018b). Attempts to directly target or block TGF-β or its pathways in the context of TAA have been unsuccessful, often exacerbating aortic pathology (Cook et al., 2015). The challenge in addressing fibrosis lies in its dual role: it serves as a physiological repair mechanism in response to trauma, but can lead to pathology when the replacement of functional tissue with scar becomes excessive (Wynn and Ramalingam, 2012; Wernig et al., 2017).

Statins, widely recognized for their cholesterol-lowering effects by targeting HMG-CoA reductase, also significantly reduce the risk of heart attacks, acute heart failure, and mortality (Zhou and Liao, 2009; Bugiardini et al., 2022). Of note, in our context, statins may effectively act as anti-fibrotic mediators following specific molecular mechanisms as recently reviewed by Dolivo et al. (2023). Atorvastatin, in particular, has been noted for its role in inhibiting pro-fibrotic gene expression and the activation of angiotensin II-induced MAPK, thereby offering protection against vascular fibrosis (Ruperez et al., 2007; Dolivo et al., 2023). Additionally, statins have been shown to counteract the activation of Rho-associated protein kinase (ROCK) by inhibiting RhoA GTPase, which helps prevent the harmful production of collagen (Akhmetshina et al., 2008; Dolivo et al., 2023). Our findings support these observations, demonstrating similar anti-fibrotic effects of statins in VSMC derived from TAA patients.

The potential therapeutic application of statins in treating TAA is supported by a growing body of *in vitro* and pre-clinical research. However, there is a notable gap in the data regarding their effectiveness as anti-fibrotic agents in preventing or limiting TAA. The literature points to various mechanisms through which statins may exert their effects on TAA. For instance, they have been shown to work alongside sartans (ARBs) to inhibit the activity of NADH/NADPH oxidase, which is implicated in TAA development (Ejiri et al., 2003). Additionally, simvastatin has been found to mitigate thoracic aorta remodeling by decreasing the secretion of cyclophilin A, downregulating its receptor CD147, and subsequently reducing the activation of the ERK1/2-cyclin signaling pathway (Tang et al., 2017). Our meta-analysis, partially filling the gap, highlights that statin could effectively acts on VSMC to prevent TAA growth with a decreased risk of adverse events.

The debate over statin therapy for patients with TAA undergoing surgery is indeed ongoing. In 2021, Kindzelski et al. (2021) reported concerns about the use of statins in this context, suggesting that caution is warranted due to a lack of conclusive data and potential risks, such as postoperative renal failure and the need for further aortic interventions or all-cause mortality after surgery. Despite these findings, the discontinuation of statins is not advised for patients pre- or post-TAA surgery, indicating the complexity of the issue and the need for more comprehensive studies to fully understand the benefits and risks of statin therapy in the surgical management of TAA (Klein and Jovin, 2021).

Our results highlight the potential of statins in managing TAA progression and improving patient outcomes. However, further research is needed i) to understand the precise anti-fibrotic mechanisms of statins, ii) to evaluate, in large-scale studies, the efficacy and safety of statins in patients with TAA, focusing on optimal dosing and duration of treatment, iii) to investigate the synergistic effects of statins with other drugs, such as ARBs, for more effective treatment protocols, and iv) to identify biomarkers to predict response to statin therapy, enabling personalized treatment approaches. Although our study supports the use of statins in the management of TAA, further research is essential to fully understand their benefits and develop evidence-based clinical guidelines.

## Limitations

The present study has limitations that should be acknowledged. A relatively small number of studies analyzed, with only four studies included for TAA growth rate and three for outcomes, which may affect the generalizability of the results. The heterogeneity among the studies and the presence of publication bias and small-study effects, despite adjustments, could also impact the robustness of the findings. Additionally, the types of statins used were not specified in all studies, except for one that used pitavastatin monotherapy, which may influence the comparability and applicability of the results. The patient demographics, with a majority being male and of a certain age range, may limit the applicability of the findings to a broader population. Moreover, the inability to analyze data separately for ascending and descending aortic segments due to study limitations further restricts the specificity of our conclusions. Of note, the study by Jovin et al. (2012) provided data on the entire aorta without specifying the location, with 2/3 of the studied population having ascending TAA. Finally, the *in vitro* component of the study, while supportive, may not fully represent the complex *in vivo* environment and the multitude of factors influencing TAA progression and outcomes.

## Conclusion

Statins, commonly known for their cholesterol-lowering effects, have shown a protective effect against the growth rate of TAA and the associated adverse outcomes, by exhibiting anti-fibrotic properties on VSMCs, which play a pivotal role in the structural integrity of the aortic wall. The anti-fibrotic effect could help in preventing the pathological remodeling of the aorta that leads to aneurysm formation.

Statin use could potentially alter the natural course of the disease, reducing the need for surgical intervention and improving the overall prognosis for patients with TAA. However, due to lack of effective drug treatments for TAA, we believe our findings highlight the need for more in-depth research to explore the potential benefits of statins in this context.

## Data Availability

The raw data supporting the conclusions of this article will be made available by the authors, without undue reservation.
