# Supplementary material for: Exploiting the anti-fibrotic effects of statins on thoracic aortic aneurysm progression: results from a meta-analysis and experimental data
Source: Front Pharmacol. 2024 Aug 1;15:1426982. doi: 10.3389/fphar.2024.1426982 (PMC11324425; doi:10.3389/fphar.2024.1426982)
Supplement: Supplementary file 1 [file DataSheet1.docx]

**SUPPLEMENTARY MATETIAL**

**
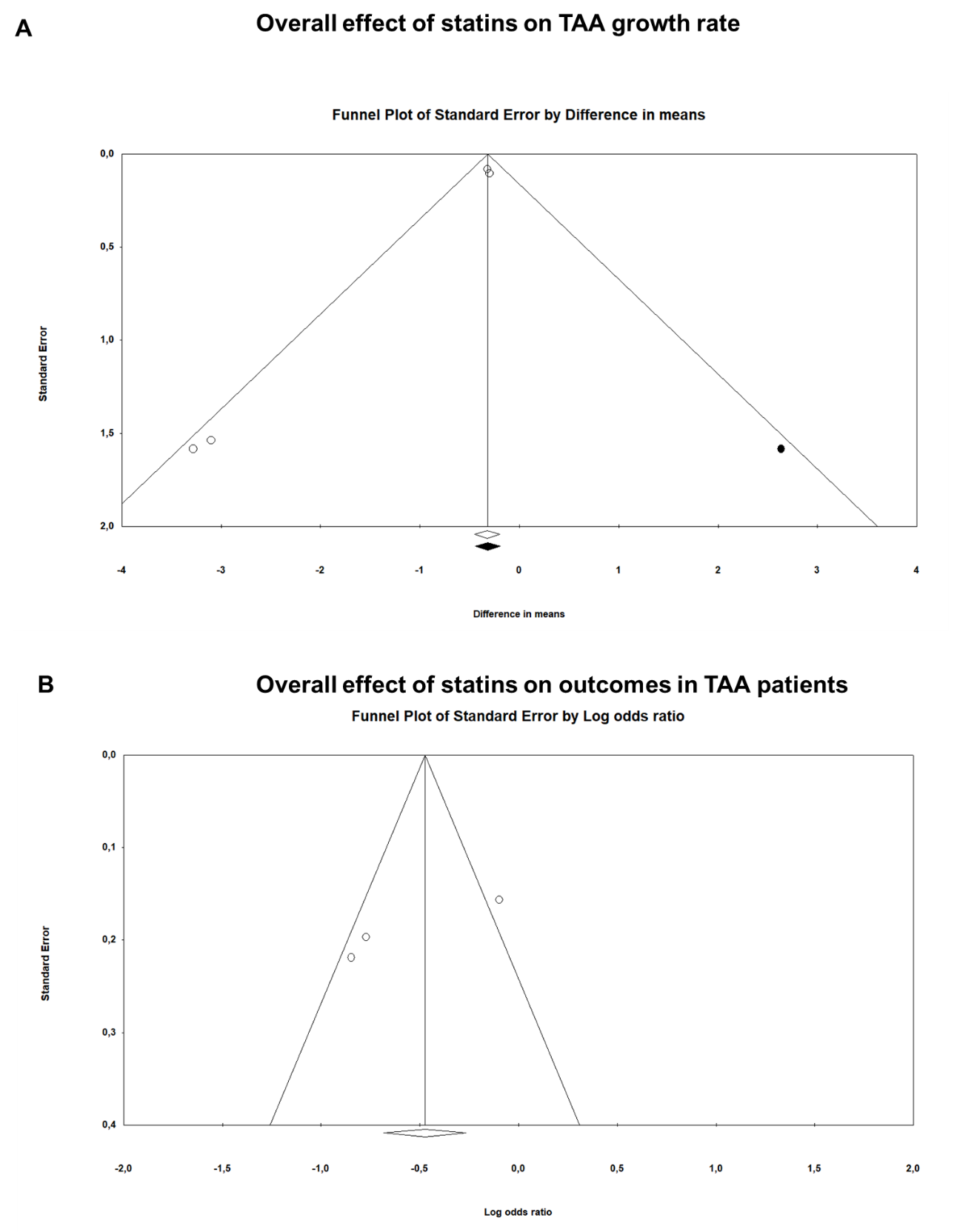
**

**Supplemental Figure S1.** (**A**) Funnel plot of effect size versus standard error for the overall effect of statins on TAA growth rate. Dots represent the single studies while diamonds are the overall standardized mean difference (**B**) Funnel plot of effect size versus standard error for the overall effect of statins on outcomes in TAA patients. Dots represent the single studies while diamonds are the overall log odds ratio. White filling refers to real studies while black fill relates to Duval and Tweedie’s trim and fill method.

**Supplementary Table S1.** **Quality assessment with Newcastle-Ottawa scale of the included studies.**

| **Author, year** | **SELECTION** | | | | **COMPARABILITY** | **OUTCOME** | | |  |
| --- | --- | --- | --- | --- | --- | --- | --- | --- | --- |
|  | **Representativeness of the Exposed Cohort** | **Selection of the Non-Exposed Cohort** | **Ascertainment of Exposure** | **Demonstration That Outcome of Interest Was Not Present at Start of Study** |  | **Assessment of Outcome** | **Enough Follow-Up Long for Outcomes to Occur** | **Adequacy of Follow Up of Cohorts** | **Quality** |
| Jovin 2012 | ** | * | * | - | * | * | ** | * | **9** |
| Stein 2013 | ** | * | * | - | * | * | * | * | **8** |
| Angeloni 2015 | * | * | * | - | * | * | * | * | **7** |
| Misaki 2018 | * | * | * | - | * | - | * | * | **6** |

**Supplementary Table S2. Characteristics of patients included in the growth rate analysis**

| **Author, year** | **Study**  **Design** | **Aorta**  **type** | **TAA**  **Statin** | **TAA**  **No Statin** | **Age,**  **overall** | **Male sex, overall** | **HP,**  **overall** | **DM,**  **overall** | **Growth rate measurement** |
| --- | --- | --- | --- | --- | --- | --- | --- | --- | --- |
| **Jovin, 2012** | Retro | all aorta | 46 | 169 | 70.2 | 62.7 | 31.8 | 5.9 | The rate of aneurysm progression was calculated using linear growth assumptions and using the instrumental variable estimation method in patients who had ≥2 imaging studies. Detailed information in “Procedures for Estimating Growth Rates in Thoracic Aortic Aneurysms” (DOI: 10.1016/S0895-4356(98)00050-X). |
| **Stein, 2013** | Retro | ascending | 231 | 710 | 62.7 | 62 | 66.7 | - | The growth rate was calculated as the linear change in the aortic diameter over time. |
| **Angeloni, 2015** | Retro | ascending | 329 | 329 | 66.3 | 60.7 | 82.4 | 16 | Treatment groups were investigated for differences in AA maximum diameter.  Echocardiographic follow-up was completed at 3 years after the first visit.  Maximum diameter of the ascending aorta was estimated by means of complete M-mode, and bi-dimensional trans-thoracic echocardiographic assessments performed with a MyLab 30 Gold Cardiovascular system (Esaote SPA, Genoa, Italy). All echocardiographic studies were reviewed in core laboratory and independently reviewed by two echocardiologists. In the case of aortic diameters greater than 45 mm, and in cases of difficult interpretation an angio-CT scan was performed and the resulting diameter was used. |
| **Misaki, 2018** | Prosp | arch | 18 | 18 | 65.5 | 83 | 77.5 | 2.75 | Contrast-enhanced CT images were reconstructed at 1 mm thickness with 1 mm intervals. Multiplaner reconstruction images were generated using ZioTerm 2009 (Ziosoft inc., Tokyo, Japan). Maximum short axis diameters were measured at cross sections perpendicular to the vascular: distal aortic arch.  These measurements were performed in a blinded manner  by two observers, one radiologist, and one cardiovascular  surgeon, and the mean value was used for the analysis. |

Retro: retrospective; Prop: prospective; TAA: thoracic aortic aneurysm; HP: hypertension; DM: diabetes mellitus

**Supplementary Table S3. Characteristics of patients included in the analysis of outcome in TAA patients**

| **Author, year** | **Study**  **Design** | **Aorta**  **type** | **TAA**  **Statin** | **TAA**  **No Statin** | **Age,**  **overall** | **Male sex, overall** | **HP,**  **overall** | **Follow-up,**  **years** | **Outcomes** |
| --- | --- | --- | --- | --- | --- | --- | --- | --- | --- |
| **Jovin, 2012** | Retro | all aorta | 147 | 502 | 70.2 | 62.7 | 31.8 | 3.6 | The composite outcome included death, rupture, dissection, or repair.  The indication for surgery was usually evidence of rupture or dissection, symptoms caused by the aneurysm, or rapid aneurysm growth or aneurysm size >5 or 5.5 cm in the ascending aorta or >6 or 6.5 cm in the descending aorta in Marfan and non-Marfan patients, respectively. |
| **Stein, 2013** | Retro | ascending | 369 | 1191 | 62.7 | 62 | 66.5 | 10 | The composite outcome included adverse events (*i.e.*, death, dissection, or rupture). |
| **Angeloni, 2015** | Retro | ascending | 329 | 329 | 66.3 | 60.7 | 82.4 | 3 | Treatment groups were investigated for differences in rates of survival free from death and/or complications,  such as dissection, rupture or needing for surgical repair. |

Retro: retrospective; Prop: prospective; TAA: thoracic aortic aneurysm; HP: hypertension
